# Human-like intelligent automatic treatment planning of head and neck cancer radiation therapy

**DOI:** 10.1088/1361-6560/ad4b90

**Published:** 2024-05-30

**Authors:** Yin Gao, Yang Kyun Park, Xun Jia

**Affiliations:** 1 Department of Radiation Oncology and Molecular Radiation Sciences, Johns Hopkins University, Baltimore, MD, United States of America; 2 Department of Radiation Oncology, University of Texas Southwestern Medical Center, Dallas, TX, United States of America

**Keywords:** deep reinforcement learning, automatic treatment planning, radiation therapy, H&N cancer

## Abstract

*Objective.* Automatic treatment planning of radiation therapy (RT) is desired to ensure plan quality, improve planning efficiency, and reduce human errors. We have proposed an Intelligent Automatic Treatment Planning framework with a virtual treatment planner (VTP), an artificial intelligence robot built using deep reinforcement learning, autonomously operating a treatment planning system (TPS). This study extends our previous successes in relatively simple prostate cancer RT planning to head-and-neck (H&N) cancer, a more challenging context even for human planners due to multiple prescription levels, proximity of targets to critical organs, and tight dosimetric constraints. *Approach.* We integrated VTP with a real clinical TPS to establish a fully automated planning workflow guided by VTP. This integration allowed direct model training and evaluation using the clinical TPS. We designed the VTP network structure to approach the decision-making process in RT planning in a hierarchical manner that mirrors human planners. The VTP network was trained via the *Q*-learning framework. To assess the effectiveness of VTP, we conducted a prospective evaluation in the 2023 Planning Challenge organized by the American Association of Medical Dosimetrists (AAMD). We extended our evaluation to include 20 clinical H&N cancer patients, comparing the plans generated by VTP against their clinical plans. *Main results.* In the prospective evaluation for the AAMD Planning Challenge, VTP achieved a plan score of 139.08 in the initial phase evaluating plan quality, and 15 min of planning time with the first place ranking in the adaptive phase competing for planning efficiency while meeting all plan quality requirements. For clinical cases, VTP-generated plans achieved an average VTP score of $125.33\pm11.12$, which outperformed the corresponding clinical plans with an average score of $117.76\pm13.56$. *Significance.* We successfully integrated VTP with the clinical TPS to achieve a fully automated treatment planning workflow. The compelling performance of VTP demonstrated its potential in automating H&N cancer RT planning.

## 1. Introduction

Treatment planning plays an increasingly important role in modern cancer radiation therapy (RT). In advanced treatment techniques, such as intensity-modulated RT (IMRT) and volumetric modulated arc therapy (VMAT), thousands of parameters are determined via a treatment planning process to control the operation of a medical linear accelerator (LINAC), such as its gantry angles, positions of multi-leaf collimators, and delivered monitor units (MUs), to precisely guide the LINAC to deliver a highly sculptured 3D dose distribution conformal to the tumor target, while maximally sparing radiation dose to normal organs. This step is often accomplished by solving an inverse optimization problem using a treatment planning system (TPS). Specifically, a treatment planner operates the TPS to define a set of dosimetric constraints for the optimization problem. The TPS then launches its optimization engine to determine a solution for the constraints. Based on the solution, the planner manually refines the constraint parameters to steer the solution towards a plan meeting the desired plan quality. It is well known that this trial-and-error treatment planning process causes plan quality variations that highly depend on factors such as the available time for planning and the planner’s experience (Nelms *et al* 2012, Hernandez *et al* 2020). Current human-centered treatment planning also poses a laborious and time-consuming workflow, presenting challenges in time-sensitive scenarios such as adaptive radiotherapy that requires frequent and rapid planning (Yan *et al* 1997, Li *et al* 2013, Brock 2019, Glide-Hurst *et al* 2021). To mitigate these challenges, automatic planning strategies have been proposed as a solution (Tol *et al* 2015, Hussein *et al* 2018).

Over the years, extensive research has been conducted to develop techniques that can automate the treatment planning process. Novel approaches have been employed, such as greedy algorithms (Xing *et al* 1999, Wu and Zhu 2001), inverse optimization (Babier *et al* 2018), knowledge-based planning (Lee *et al* 2013, Ge and Wu 2019), and multi-criteria optimization (Craft *et al* 2012, Zarepisheh *et al* 2014, Breedveld *et al* 2019). Recently, deep learning (Shan *et al* 2020, Shen *et al* 2020b) has also demonstrated its power in this domain, for instance by predicting patient-specific best achievable dose distribution to guide treatment planning (Chen *et al* 2019, Nguyen *et al* 2019, 2020).

One particular approach for automated treatment planning is based on reinforcement learning (RL), or its modern version, deep RL (DRL) that couples RL with the recent advances in deep learning. In RL, a computer agent is trained to be able to autonomously make decisions in response to the observed state of the environment. Specific to the treatment planning context, a virtual treatment planner (VTP) can be trained to operate the TPS, in lieu of a human planner, to define and refine dosimetric constraints and generate high-quality plans (Shen *et al* 2019, Hrinivich and Lee 2020, Zhang *et al* 2021, Sprouts *et al* 2022). Within this framework, we have successfully developed the Intelligent Automatic Treatment Planning (IATP) framework. Our systematic work in this domain has not only demonstrated its potential in external beam RT and brachytherapy (Shen *et al* 2019, 2020a) but also overcome several practical challenges to support clinical applications, such as building large models for sequential decision-making (Shen *et al* 2021b) and enhancing training efficiency by incorporating prior information (Shen *et al* 2021a). In recent work, we successfully translated the development and evaluated its performance in real clinical cases of prostate cancer RT, demonstrating the achievement of treatment planning capability of VTP comparable to human planners with a slightly higher mean plan quality score (Gao *et al* 2023). In a retrospective, but objective evaluation context of the prostate stereotactic body RT (SBRT) Planning Challenge organized by the American Association of Medical Dosimetrists (AAMD) in 2016 (American Association of Medical Dosimetrists 2023), our VTP achieved performance at third place compared to human planners who participated in this challenge with IMRT technique.

Along with these successes, we continued our development of IATP by extending it to head-and-neck (H&N) cancer RT treatment planning, which is the focus of this study. Specifically, we successfully developed a VTP to accomplish a fully automated planning workflow for H&N cancer RT by having it operate a commercial TPS in lieu of a human planner. The VTP was tailored for the 2023 AAMD Planning Challenge that focused on not only generating a high-quality treatment plan for an H&N cancer case but also on the most efficient planning process to address the need for online adaptive RT. Our VTP participated in the Planning Challenge and achieved the first position in planning efficiency while meeting all dosimetric requirements. This paper will also report a comprehensive evaluation of clinical cases, where we compared the quality of plans generated by the VTP against those generated by experienced human planners.

## 2. Materials and methods

### 2.1. Overview of VTP and treatment planning workflow

#### 2.1.1. VTP

VTP is a neural network trained to operate the TPS, in lieu of a human planner, to define and refine dosimetric constraints and generate high-quality plans. Our group previously introduced a hierarchical VTP (HieVTP) to address the treatment planning task, emulating the hierarchical decision-making used by human planners (Shen *et al* 2021b). HieVTP was structured into three sub-networks: Structure-Net, Parameter-Net, and Action-Net, serving the roles of making decisions at various levels—structure, parameter, and adjustment action. They were sequentially applied each time HieVTP interacted with the TPS. Specifically, Structure-Net utilized the dose-volume histograms (DVHs) of the plan to identify structures in need of improvement. Subsequently, Parameter-Net selected a treatment planning parameter (TPP, e.g. priority, dose limit, or volume constraint) that had the most impact on the dose of the structure, considering all associated TPPs. Finally, Action-Net determined the specific adjustment for this TPP, which entailed increasing or decreasing its value.

In this study, for the purpose of improving training efficiency, we reduced the complexity of this HieVTP in two aspects. The first one was combining Structure-Net with Parameter-Net. The Parameter-Net decided on one of the eight TPPs to adjust, and subsequently, the Action-Net determined its adjustment direction.

Another aspect was to input the VTP with a vector containing plan quality scores, as opposed to the DVHs in our previous studies, because these scores directly indicate plan quality, making it straightforward for the VTP to extract relevant information to make decisions for plan quality improvement. The list of scores followed the ProKnow scoring system (ProKnow Systems, Elekta, Sanford, FL, USA), as we applied our VTP to the 2023 AAMD Planning Challenge that employed this metric for plan quality (American Association of Medical Dosimetrists 2023). The ProKnow scoring system is a commercial software product designed to offer a fair platform for evaluating plan quality. It is a sum of a number of scores, each representing plan quality from a certain aspect (Nelms *et al* 2012). For the context of H&N planning, the evaluation criteria on plan quality are listed in the supplement material (table S1).

**Figure 1.**
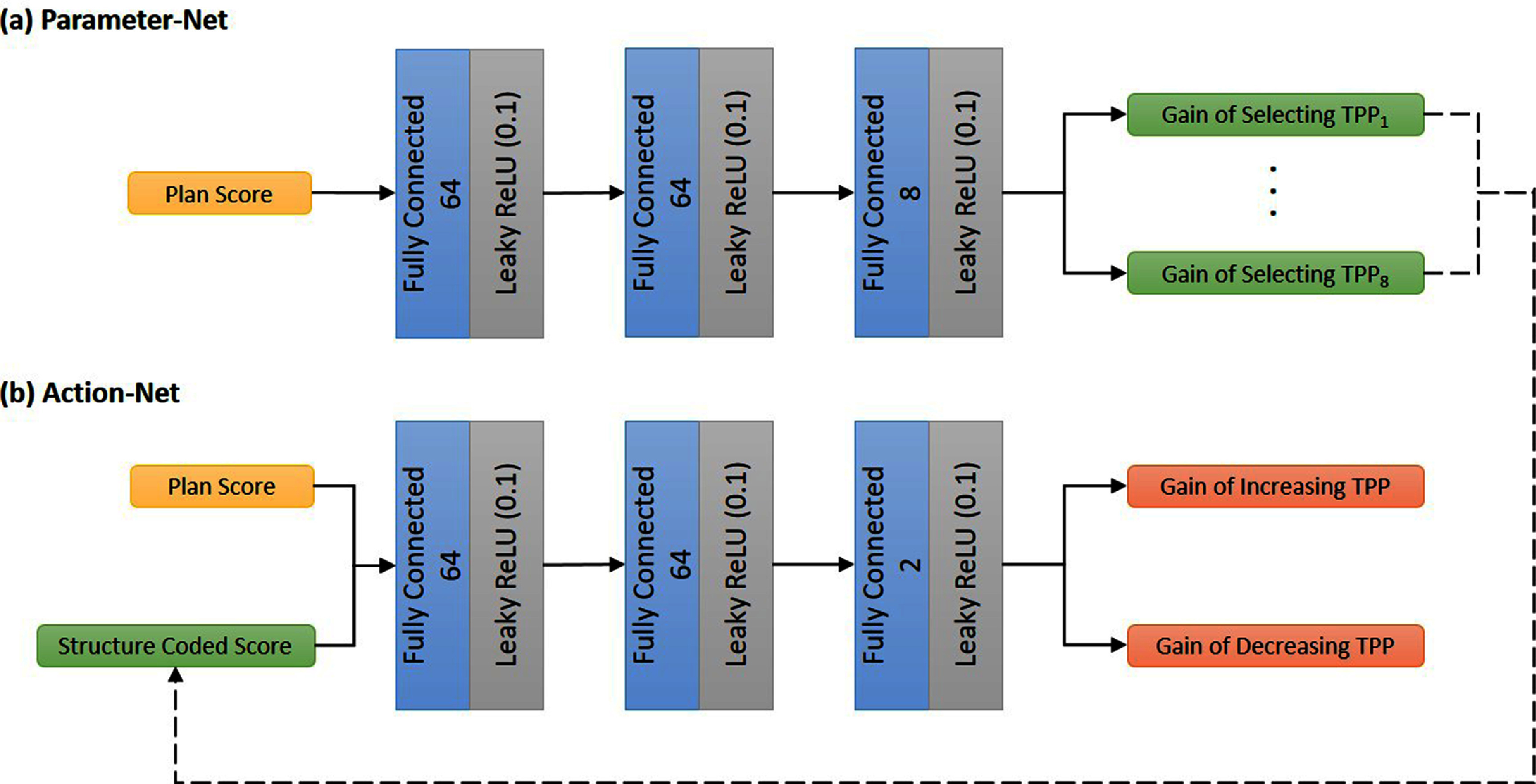
The detailed architecture of the VTP. VTP consists of two subnetworks (a) Parameter-Net and (b) Action-Net. Output sizes for the fully connected layers are specified.

With these considerations in mind, the input to the VTP consisted of 21 values, corresponding to various dosimetric criteria. Both sub-networks process data through a sequence of three fully connected layers, with each layer followed by a Leaky ReLU action function with a slope of 0.1. Following the final activation function, Parameter-Net predicted the improvement in plan quality associated with adjusting the TPPs of each structure. This prediction informed the creation of a structure-coded vector, which served as an indicator for the selected structure. The structure-coded vector maintained the specific score of the chosen structure while setting all other scores to zero, preserving the same dimension as the original input. Subsequently, this vector was concatenated with the original 21 ProKnow scores. The combined vector was then passed into the Action-Net to predict the improvement in plan quality corresponding to each parameter adjustment action. The detailed architecture of the VTP is presented in figure 1. Each parameter was allowed for two adjustment directions (increasing or decreasing). The adjustment magnitude for TPPs was empirically selected. We opted to increase the priority value by 6 if there was an improvement over an empirically chosen criterion of 20% in the plan score during the last TPP adjustment or reduce the adjustment size to 3 if the plan score change was less than 20%, deemed minor.

#### 2.1.2. Auto-planning workflow

**Figure 2.**
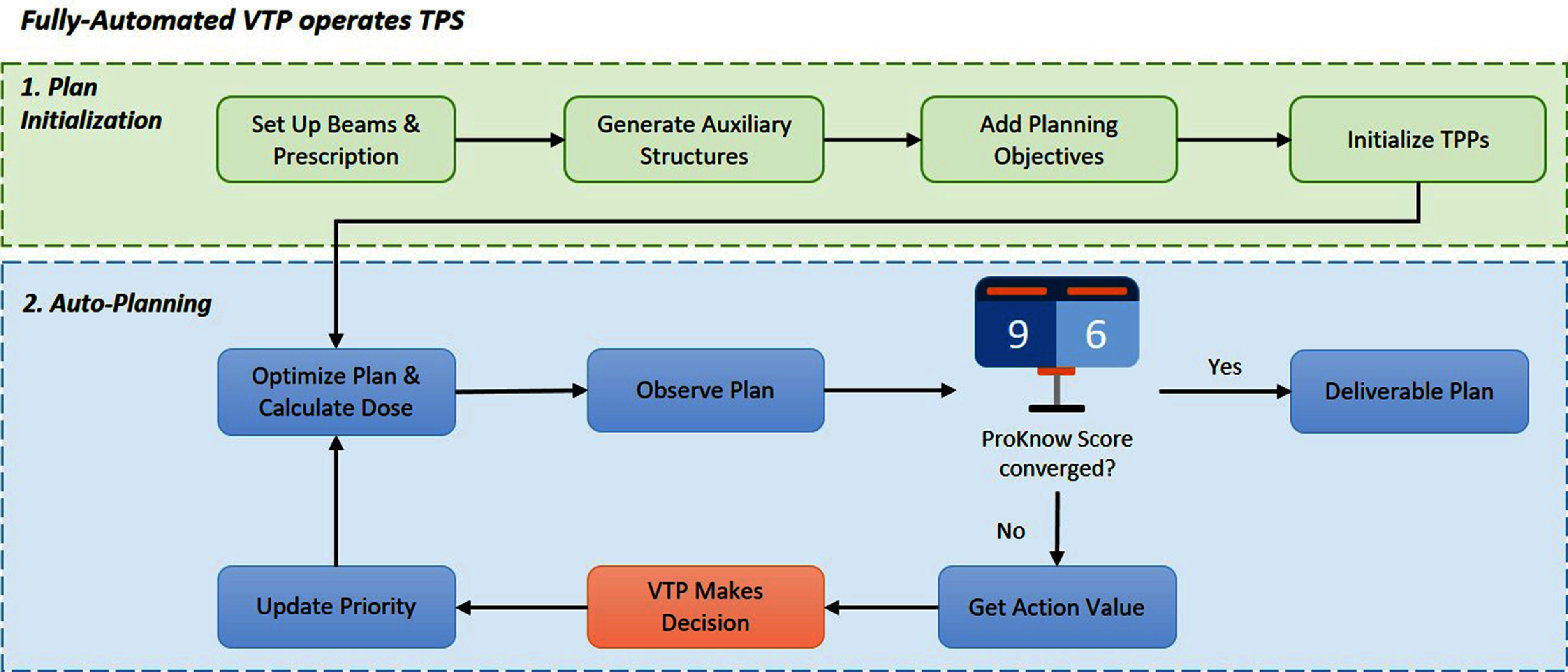
The fully automated workflow of VTP to automatically generate VMAT plan for H&N cancer cases in Eclipse TPS.

Figure 2 outlines the automated workflow of VTP for H&N cancer RT treatment planning within the Eclipse TPS (Varian Medical System, Palo Alto, CA, USA). The treatment planning process by the VTP was initiated through the Eclipse Scripting Application Programming Interface (ESAPI). It first autonomously configured the treatment planning initialization process, including tasks such as specifying the isocenter, defining beam fields, prescribing the dose, and creating auxiliary structures that aid in shaping the dose distribution. Following this, VTP introduced TPPs, such as dosimetric objectives for each structure to formulate the optimization problem within the TPS. Plan optimization and dose calculations were then carried out in the background. Once the optimization phase produced a plan, VTP collected the plan data and evaluated the plan quality. In the case that the stopping criteria were not met, VTP proceeded to determine adjustments to the TPPs for the subsequent planning iteration and start a new optimization cycle. This iterative planning process continues until a high-quality plan is achieved.

### 2.2. Training the VTP

#### 2.2.1. RL

The training process of the VTP followed the *Q*-learning framework (Watkins and Dayan 1992). We present it briefly here for completeness. Readers interested in details can refer to our previous publications (Shen *et al* 2021b).

In *Q*-learning, the training process determines the optimal action-value function

\begin{align*} Q^*\left(s,a\right) = \max_{\pi}\left[r^l+{\gamma}r^{l+1}+{\gamma}^2r^{l+2}+\cdots|s^l = s,a^l = a,{\pi}\right],\end{align*}

where $Q^*(s, a)$ indicates the expected cumulative rewards associated with taking action *a* (the decision related to TPP adjustment policy) in state *s* (represented by the vector of ProKnow scores). It quantifies the desirability of action *a* in the context of state *s*. *s^l^* and *a^l^* represent the state and action at the *l*th TPP adjustment step, respectively. *r^l^* denotes the reward obtained at the *l*th step, which is determined by a predefined reward function related to clinical objectives, such as the ProKnow scoring system. A positive reward is achieved when the action applied to the state leads to improved plan quality with the updated TPP. The term $\pi = P(a|s)$ refers to the policy of TPP adjustments, signifying the selection of action *a* based on the observed state *s*.

In DRL, the action-value function $ Q^*(s,a)$ is represented by deep neural networks as described in the previous subsection. It is obtained by a training process including an iterative process based on the VTP’s experiences with TPP adjustments and interactions with the TPS. The primary goal of training is to determine the optimal policy for VTP to adjust the TPP of the selected structure to maximize the expected cumulative reward. The update of the *Q*-learning action-value function can be expressed in the following manner, where *α* is the learning rate to control the weight given to the new information obtained from the current experience.

\begin{align*} Q^*\left(s,a\right) \leftarrow Q^*\left(s,a\right)+\alpha\left[r^l+\gamma\max\left(Q\left(s^l,a^l\right)\right)-Q\left(s,a\right)\right].\end{align*}

#### 2.2.2. Reward function

A prerequisite for VTP is to quantitatively define H&N cancer plan quality. In this study, we incorporated the ProKnow scoring system, which served as the plan evaluation criteria during the 2023 AAMD Plan Study. ProKnow scoring system includes a series of dosimetric metrics of the structure of interests (American Association of Medical Dosimetrists 2023). The detailed computation methods of all metrics were referred to the planning guidance provided by AAMD, which can be found in supplementary material table S1. The final score $\psi(d)$ as a function of the dose distribution of a plan *d* was determined by the summation of the scores across all these metrics, resulting in a plan score that ranges from 0 to 150, where a higher score corresponds to superior plan quality.

Based on the plan quality score, the reward function to guide the learning process in DRL was naturally defined as the change in plan quality for a TPP adjustment, namely $r = \psi(d^{^{\prime}}) - \psi(d)$, where dose *d* and *d*′ are dose distributions before and after the TPP adjustment. A positive reward signifies an enhancement in plan quality, whereas a negative reward indicates the opposite.

#### 2.2.3. DRL training strategy

Our previous work has established the basis for training with hierarchical DRL by incorporating a multi-level decision-making hierarchy that enabled VTP to learn and strategize across multiple levels. We employed the same training strategy with modifications to adapt to the two-level decision-making process of VTP in this study.

Let’s denote Parameter-Net as $S(s,p;\theta^*_S)$ and Action-Net as $A(s,p,a;\theta^*_A)$. Here, $\theta_S^*$ and $\theta_A^*$ represent the optimal network parameters to be determined from the training process, *s* is the state of the treatment plan (21 ProKnow scores) to input to the networks, *p* is the set of TPP to adjust, and *a* the action to adjust the TPP. Once $S(s,p;\theta^*_S)$ is known, the policy to decide the TPP $p^*$ to adjust for the plan state *s* is achieved by the one that maximizes it, i.e. $p^* = \max_pS(s,p;\theta^*_S)$. After that, based on the selected TPP, the Action-Net then decides on one of two actions $a^*$ to adjust by selecting the one that maximizes the function, i.e. $a^* = \max_aA(s,p^*,a;\theta^*_A)$.

In terms of training, we alternatively trained the two networks $S(s,p;\theta_S)$ and $A(s,p,a;\theta_A)$, each time fixing one while training the other. When $S(s,p;\theta^*_S)$ is fixed, $A(s,p,a;\theta_A)$ can be updated with the standard Q-learning algorithm to solve the problem

\begin{align*} \min_{\theta_A}\left[\|A\left(s,p,a;\theta_A\right)-\left(r+\gamma \max S\left(s^{^{\prime}},a;\theta_S\right)\right) \|^2_2\right].\end{align*}

With the updated $A(s,p,a;\theta_A)$ fixed, $S(s,p;\theta_S)$ can subsequently be trained as a conventional supervised learning problem by solving

\begin{align*} \min_{\theta_S}\left[\|S\left(s,p;\theta_S\right)-\max_p A\left(d,p,a;\theta_A\right) \|^2_2\right].\end{align*}

We trained the VTP based on the single patient case in the 2023 AAMD Planning Challenge. Throughout the training process, VTP engaged with the Eclipse TPS for treatment planning and collecting data for training. At each iteration of TPP adjustment, we incorporated an *ε*-greedy algorithm to introduce stochasticity. This involved VTP making a random selection among all possible actions with a probability of *ε*, and a probability of $(1-\epsilon)$ to choose actions based on VTP’s learned strategies. The value of *ε* gradually diminished at a rate defined as $\epsilon = \max(0.1, 0.99/\mathrm{EPI})$, where $\mathrm{EPI}$ is the index of episodes. The *ε*-greedy strategy allowed VTP to explore diverse state-action pairs without biasing towards prior experiences. In each training iteration, a tuple $(s, a, r, s^{^{\prime}})$, including the state *s*, action *a*, reward *r*, and the next state *s*′, was collected and stored in a pool. Experience replay strategy was employed to sample data from this pool to update network parameters. Random sampling ensured uncorrelated experiences for updating Q-values, reduced the risk of overfitting, and encouraged exploration, allowing VTP to learn from both successes and failures and refine its strategies over time.

It is worth noting that, in our previous developments on prostate SBRT (Shen *et al* 2021b), we built the VTP trained with an in-house TPS with an inverse planning optimization engine similar to Eclipse TPS. This approach was chosen due to the inefficiencies associated with direct VTP interaction with Eclipse TPS during the training process. We successfully trained the VTP under this setup, which was able to autonomously create high-quality treatment plans using the Eclipse TPS. However, when it comes to the H&N cancer case, we observed the limitation of this approach due to slight dose disparities between the two TPSs. To address these issues, in the current study, we seamlessly integrate VTP with Eclipse TPS, facilitating direct training using it. As such, the state *s* and *s*′, as well as the reward *r*, were all computed based on dose distribution computed by the Eclipse dose engine, mitigating the issue of mismatch in dose calculations between the TPS used in training and application of VTP.

The VTP was constructed using Python with Pytorch on a desktop workstation with two Nvidia Quadro RTX 5000 GPUs on a computer equipped with a CPU of 26 cores and 64 GB of host memory. We interfaced the VTP with Eclipse TPS v16.0 using ESAPI to enable the fully automated planning workflow and collection of training data.

### 2.3. Configuration of plan optimization problem

Because the auto-planning workflow directly interacts with the Eclipse TPS, the plan optimization problem was indeed the one in the Eclipse TPS. To define the objective function, a series of dosimetric constraints were specified on DVHs, each including a weighting factor, the dose of the constraint, type (dose volume constraint or mean dose), as well as the direction (upper or lower to penalize overdose or underdose). Treatment targets, such as the planning target volumes (PTVs), have both upper and lower objectives in planning to ensure both dose coverage and dose homogeneity. In contrast, organs at risk (OARs) typically have upper dose objectives, with the primary aim of minimizing radiation exposure to these critical structures.

In H&N cancer treatment planning, challenges arose due to significant overlap between targets and OARs. To achieve a sophisticated dose distribution, human planners often create auxiliary structures by cropping the regions of OARs that overlap with targets and introducing additional planning objectives for these auxiliary structures. Our automated treatment planning workflow employed this approach.

As such, we included 26 planning structures, including targets, OARs, and their auxiliary structures. The detailed list of planning structures is presented in table 1. The contours of PTVs, clinical target volumes (CTVs), and OARs were segmented by experienced radiation oncologists and dosimetrists. The target volumes were assigned prescription doses at four levels: 63 Gy, 60 Gy, 57 Gy, and 54 Gy, as indicated in their names. To facilitate effective planning, auxiliary structures were created. These included ‘$\text{L} \;\text{Parotid}_\text{opti}$’, ‘$\text{R}\;\text{Parotid}_\text{opti}$’, ‘$\text{Oral}\; \text{Cavity}_\text{opti}$’ and ‘Constrictor_opti_’, all created by excluding the overlap between each organ and the PTVs. This strategy was implemented to resolve conflicting planning objectives between targets and OARs. The ‘$Cord+5mm$’ structure was a 5 mm expansion around the spinal cord, designed to limit the maximum dose received by the cord. Additionally, ‘PTV54_Push_’ and ‘PTV57_Push_’ were segmented as 3 mm inner rings of PTV54 and PTV57, respectively, aiming to ensure sufficient dose in the peripheral regions of these target volumes. Control regions named ‘$Avoidance1$’, ‘$Avoidance2$’, and ‘$Avoidance3$’ were used to avoid dose distribution in the neck, oral cavity, and trachea. The design of these structures was determined by experienced dosimetrists. All these auxiliary structures were efficiently and automatically generated in Eclipse TPS by the VTP via ESAPI in approximately 5 seconds.

**Table 1.** Planning objectives involved in the H&N cancer planning by VTP. The first section lists the objectives fixed in our study. The second section includes objectives that VTP adjusts their priorities.

Structure	Dose (cGy)	Volume (%)	Priority	Objective type
PTV63	6330	99.9	90	Lower
	6380	98	90	Lower
PTV60	6030	99.9	90	Lower
	6080	98	90	Lower
	6480	0	100	Upper
PTV57	5750	99.9	95	Lower
	5770	99	95	Lower
	5800	98	95	Lower
	6270	0	100	Upper
PTV54	5450	99.9	95	Lower
	5470	99	95	Lower
	5500	98	95	Lower
	5970	0	100	Upper
CTV63	6330	99.9	90	Lower
	6350	99	90	Lower
	6660	0	87	Upper
CTV60	6030	99.9	90	Lower
	6050	99	90	Lower
	6500	0	100	Upper
CTV57	5750	99.9	93	Lower
	5770	99	93	Lower
	6270	0	100	Upper
CTV54	5450	99.9	95	Lower
	5470	99	95	Lower
	5940	0	100	Upper
PTV57$_\mathrm{push}$	5800	99.9	98	Lower
PTV54$_\mathrm{push}$	5500	99.9	98	Lower
PTV$_\mathrm{all}$	6500	0	80	Upper
PTV-CTV63	6500	0	80	Upper
Brainstem	2400	0	60	Upper
Cord+5 mm	2600	0	63	Upper
Esophagus	1670		65	Mean
Avoidance1	5500	0	80	Upper
	3750		30	Mean
Avoidance2	5500	0	80	Upper
Avoidance3	5700	0	100	Upper
L Parotid	3000	0	P1	Upper
	2750		P2	Mean
L Parotid$_\mathrm{opti}$	1850		P2	Mean
R Parotid	3000	0	P3	Upper
	2750		P4	Mean
R Parotid$_\mathrm{opti}$	1250		P4	Mean
Oral Cavity$_\mathrm{opti}$	2700		P5	Mean
Constrictor	5650	0	P6	Upper
	3600		P7	Mean
Constrictor$_\mathrm{opti}$	3400		P7	Mean
Brachial Plexus	6000	0	P8	Upper

As a result, we established 47 planning objectives, including 20 lower dose-volume constraints, 18 upper dose-volume constraints, and 9 mean dose constraints. Each objective *i* was specified by parameters of dose limit *τ*_*i*_, volume ${V}_{i}$ if needed for dose-volume constraints, and the weighting factor *λ*_*i*_ reflecting the priorities of the respective structure in the plan. This amounted to a total of 141 TPPs. It was computationally challenging for VTP to learn the policy for adjusting all TPPs, and neither was this necessary, as a number of these TPPs can be determined *a priori* based on human experience. As such, we held constant TPP values of 36 planning objectives designed by the planning expertise of a dosimetrist. For the remaining 11 planning objectives, the dose, volume, and type were predefined, and VTP was trained to adjust their priorities. Among the 11 adjustable TPPs, 3 TPPs of the auxiliary structures shared the same priorities as their primary planning structures, specifically parotid glands and pharyngeal constrictor. In total, the 8 TPPs define the optimization problem, which will be adjusted by the VTP. A comprehensive list of all the planning structures and objectives is summarized in table 1.

### 2.4. Evaluation

#### 2.4.1. 2023 AAMD planning challenge case

Our VTP participated in the 2023 AAMD Planning Challenge. Organized by the AAMD, a two-phase plan study was undertaken in 2023 for medical dosimetrists to develop an adaptive RT treatment plan for a patient with H&N cancer (American Association of Medical Dosimetrists 2023). This patient, a 34-year-old male, was diagnosed with poorly differentiated squamous cell carcinoma of the left retromolar trigone and positive lymph nodes. The patient’s treatment journey began with surgery, followed by post-operative concurrent chemo/RT.

During the RT course, the patient received external beam treatment with a 6 MV photon beam, and the prescription dose was 210 cGy per fraction, totaling 30 fractions. The contours were created by the physicians and dosimetrists at Mary Bird Perkins Cancer Center. The treatment plan included four levels for different PTVs at 63 Gy, 60 Gy, 57 Gy, and 54 Gy with the aim of achieving at least 95% coverage for PTV 63 Gy and PTV 60 Gy, and at least 90% coverage for PTV 57 Gy and PTV 54 Gy. This patient presents a substantial overlap between targets and OARs, which introduces complexities in the planning process. Specifically, PTV 54 Gy intersects with the oral cavity, pharyngeal constrictor, both parotid glands and esophagus. PTV 57 Gy exhibits overlap with the pharyngeal constrictor, left parotid gland, and brachial plexus. PTV 60 Gy overlaps with the oral cavity, left parotid gland, and pharyngeal constrictor. Finally, PTV 63 Gy shows an overlap with the pharyngeal constrictor and brachial plexus.

**Figure 3.**
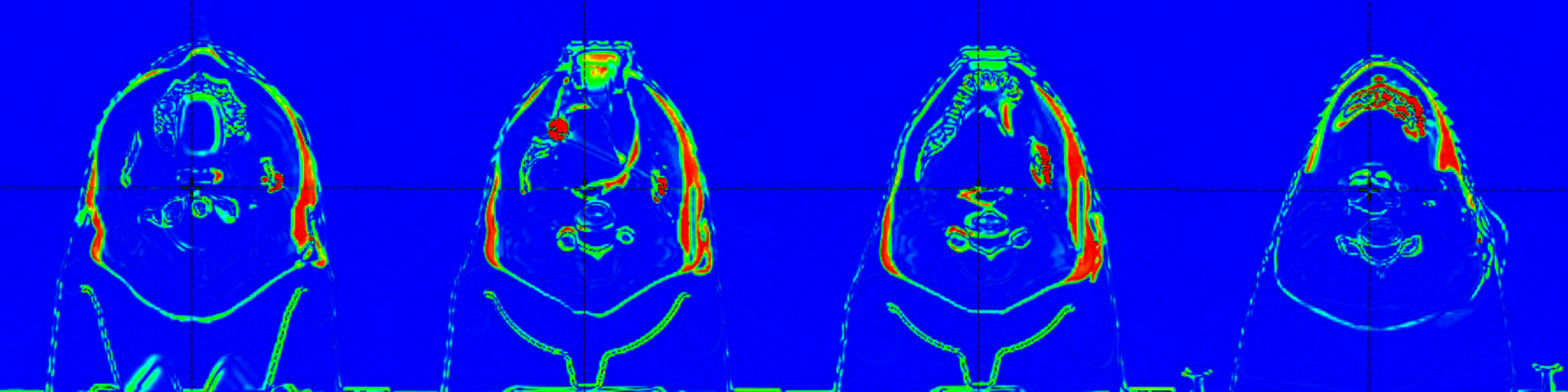
The patient experienced a 16.5% weight loss during the 4th week of RT treatment, leading to a significant change in body shape. To visualize these changes, we superimposed the initial and adaptive planning CT scans and showed four axial levels here, with the red region highlighting significant anatomical changes.

Notably, as shown in figure 3, by Week 4 of the RT course, the patient experienced a significant weight loss of 16.5%, which led to an increase of 2 cm in the source-to-surface distance on the patient’s left side and 0.8–1 cm on the patient’s right side. In response to this change, the RT team decided to implement an adaptive treatment plan, which necessitated acquiring a new CT scan and adjusting the PTVs. Hence, the purpose of the Planning Challenge was twofold. The first was to develop a high-quality plan for the initial anatomy, and the second was to, based on this knowledge, to develop an adaptive plan for the changed anatomy to meet dosimetric requirements in an efficient manner. The plan quality in this Challenge was evaluated using the ProKnow scoring system presented in section 2.2.2.

We assessed the planning performance of the developed VTP on the both initial and adaptive phases as part of the AAMD Planning Challenge. As we were participating in the Planning Challenge, we predefined a set of reasonable TPPs for VTP as the initialization of the TPP adjustment process. For the adaptive plan, we used the TPPs of the final plan in the initial phase as the starting point. Planning time was measured as the active time spent on planning operations from beam placement to plan completion.

#### 2.4.2. Comprehensive evaluations

In order to further gauge the VTP’s applicability and generalizability to managing clinically realistic cases, we extended our testing to include 20 clinical cases for patients who had undergone H&N cancer RT treatment at our institution between 2018 and 2023. Patient characteristics are listed in supplementary material table S2. The patient cohort was chosen primarily based on the prescription dose of 63 Gy in 30 fractions, following the setting in the AAMD planning challenge case. We did not consider primary tumor location or extension in patient selection. For each case, we used VTP to generate a plan. Unlike the AAMD case that included four dose levels (63, 60, 57, and 54Gy), patients at our institution are typically treated with 2–3 target dose levels. Other than the highest dose (63Gy) to the primary tumor, target dose levels may be similar to, but not exactly match, the AAMD case. For a target with different prescription dose level from the AAMD case, the fixed optimization parameters in table 1 with the closest prescription dose were chosen. For a fair comparison, each plan generated by VTP was normalized to achieve the same PTV coverage as their respective clinical plans. In our analysis, we compared the ProKnow scores between the plans generated by VTP and the scores from the clinical plans. Our assessment of plan scores and dosimetric metrics utilized a non-inferiority test with the null hypothesis that VTP is inferior to human planners at a significance level of $p^* = 0.05$. We also assessed the planning time of VTP, measuring its active operation duration from beam placement to plan completion. Additionally, we offered insights into the decision-making capabilities of VTP by contrasting the plan quality in its initial, intermediate, and final steps along the treatment planning process, thus highlighting the enhancements made during the process.

## 3. Results

### 3.1. 2023 AAMD plan study case

#### 3.1.1. Initial phase

**Figure 4.**
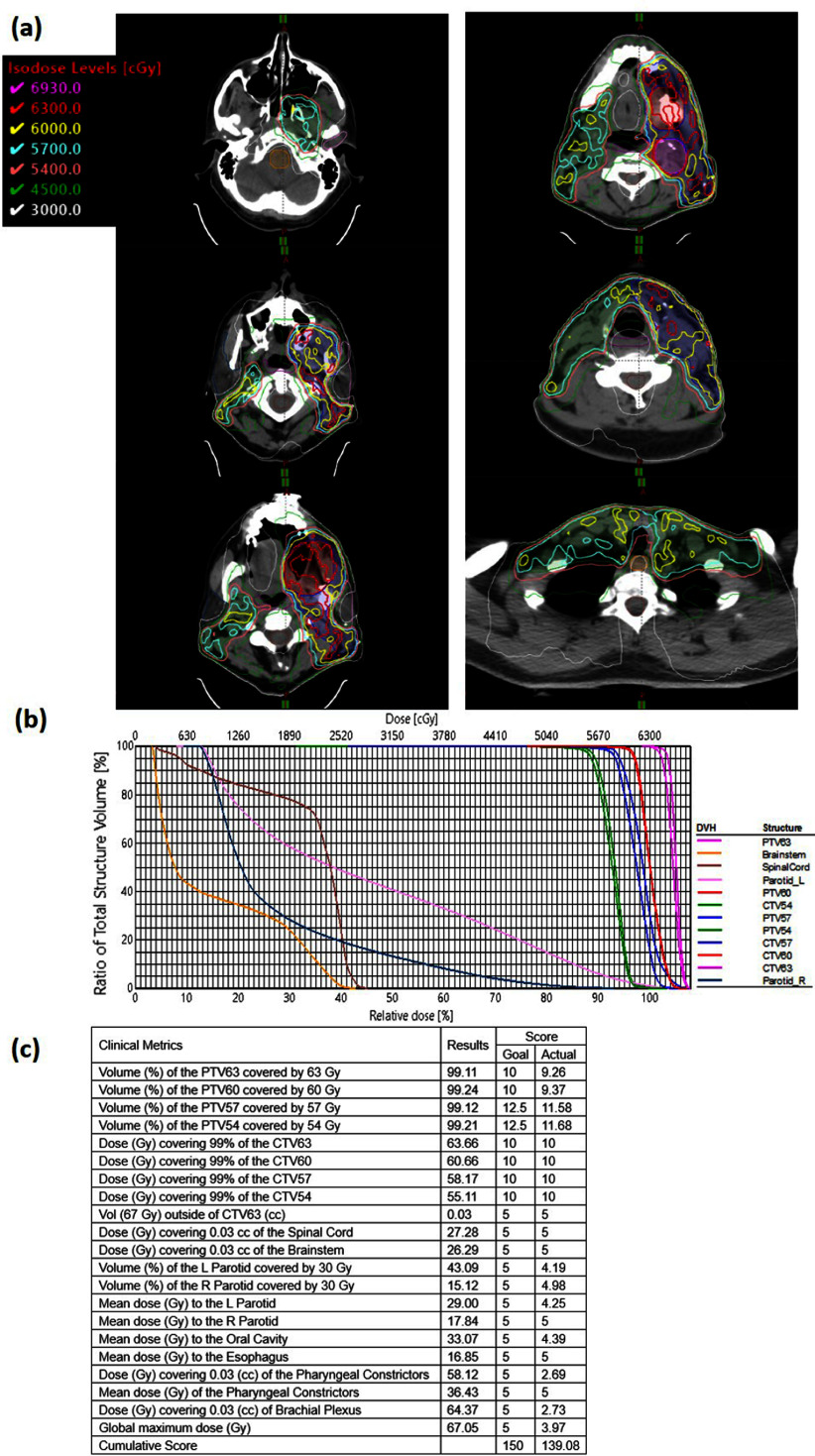
The plan for the initial phase of the AAMD Planning Challenge case. (a) Dose distributions across six axial slices from the top to the bottom of the targets. (b) DVHs. (c) Dosimetric results and scores.

VTP successfully generated a high-quality H&N cancer VMAT plan for the initial phase of the 2023 AAMD Plan Study, utilizing Eclipse TPS. Figure 4(a) presents the dose distributions across six axial slices from the top to the bottom of the targets and figure 4(b) displays the DVHs of the resulting plan. Remarkably, the VTP effectively balanced PTV and OARs during the plan optimization process as the plan maintained PTV coverage and spared the dose to OARs. For a comprehensive overview of the final VTP-generated plan, we presented the ProKnow scores in figure 4(c). The VTP-generated plan achieved full compliance with all clinical metrics outlined with a cumulative score of 139.08 out of 150. Our VTP plan was ranked the 21^*st*^ among 149 human-submitted plans with scores 127.32 ± 13.73 (American Association of Medical Dosimetrists 2023). This achievement demonstrated VTP’s competence in treatment planning, particularly considering the complexity of the H&N cancer case, where human planners can employ more auxiliary structures to refine dose distribution.

#### 3.1.2. Adaptive phase

**Figure 5.**
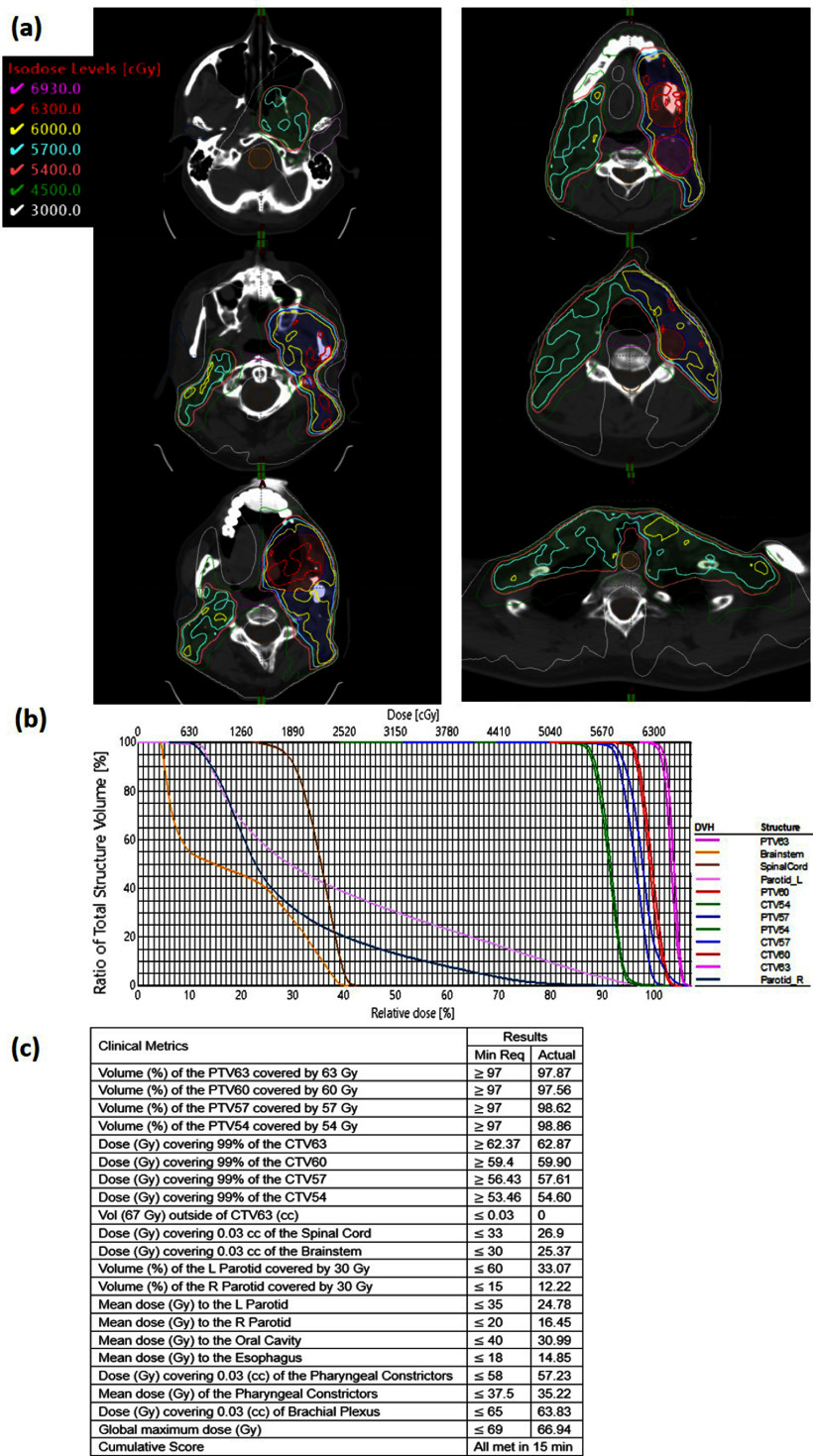
The plan generated by the VTP for the adaptive phase of the AAMD Planning Challenge case. (a) Dose distributions across six axial slices from the top to the bottom of the targets. (b) DVHs. (c) Dosimetric results and planning time.

During the RT treatment course, the patient’s substantial weight loss necessitated a reevaluation and adaptation of the treatment plan to accommodate the anatomical changes. Figure 5 presents dose distributions in six axial and DVHs of the adaptive plan for the changed patient anatomy. Similar to the process generating the initial plan, the VTP demonstrated remarkable proficiency in preserving adequate coverage of PTVs while effectively minimizing radiation dose to OARs. The primary goal of the adaptive phase of this Challenge is to expedite the patient’s treatment with the new plan while striving to achieve the dosimetric goals with the utmost precision. The specific minimally required dosimetric metrics are detailed in figure 5(c). Notably, VTP generated a plan that fulfilled all the specified requirements within 15 minutes via a fully automated planning process. VTP achieved the $1\mathrm{st}$ position in the adaptive phase competition for the shortest planning time with plans meeting the plan quality requirements among all the participants. The average planning time of all the participants was 2.62 ± 2.24 h (American Association of Medical Dosimetrists 2023). The first author of this paper also participated in the Challenge as an AAMD-certified medical dosimetrist with 2.5 years of experience and achieved a plan meeting all requirements in 30 min of planning time.

### 3.2. Clinical cases

#### 3.2.1. Decision-making behaviors of VTP

**Figure 6.**
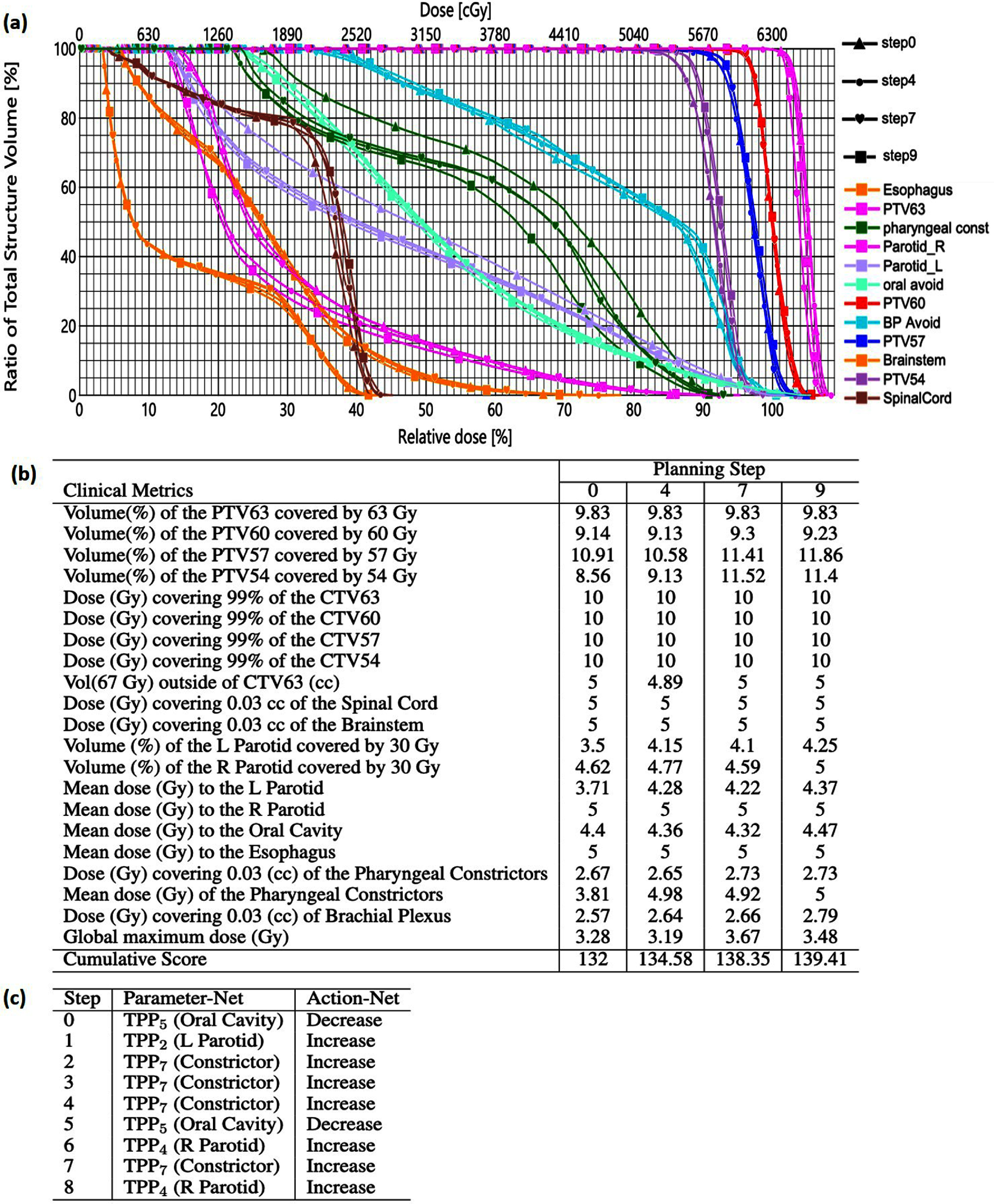
(a) DVH comparisons of plans at step 0 (triangle), 4 (dot), 7 (heart), and 9 (square). (b) The detailed scores of clinical metrics achieved by the plans at steps 0, 4, 7, and 9. (c) Decisions that VTP were made at each planning step to improve the plan score from 132 to 139.41.

We first illustrate VTP’s decision-making behaviors of operating the Eclipse TPS in a representative example case. In figure 6, we present the details of the planning process driven by the VTP to operate the TPS to improve the plan scores from 132 to 139.41 at various planning steps. Through strategic adjustments of TPPs, VTP effectively improved the scores of various metrics. For instance, by emphasizing the importance of the left parotid gland (TPP_2_), right parotid gland (TPP_4_) and pharyngeal constrictor (TPP_7_) in the plan optimization, VTP enhanced the scores of $V_\mathrm{ParotidL}[30Gy](\%)$, $D_\mathrm{ParotidL}[mean](Gy)$, $V_\mathrm{ParotidR}[30Gy](\%)$, and $D_\mathrm{Constrictors}[mean](Gy)$ by 0.75, 0.66, 0.38, and 1.19, respectively. Additionally, VTP demonstrated intelligence to make improvements by decreasing the priority of the planning structure. For example, VTP reduced the priority of the oral cavity (TPP_5_). As the oral cavity intersects with PTV volumes, this adjustment increased scores of $V_\mathrm{PTV}[54Gy](\%)$, $V_\mathrm{PTV}[57Gy](\%)$, and $V_\mathrm{PTV}[60Gy](\%)$ by 2.48, 0.95, and 0.09, without negatively impacting the score of $D_\mathrm{Oral}[\mathrm{mean}](Gy)$. Note that this entire process was autonomously completed by the VTP without human intervention. It hence indicated the decision-making capability in handling the challenging problem of H&N cancer treatment planning.

#### 3.2.2. Performance on clinical cases

To demonstrate the model’s effectiveness in treatment planning, we conducted an assessment of VTP involving 20 clinical cases of patients who received treatments at our institution.

**Figure 7.**
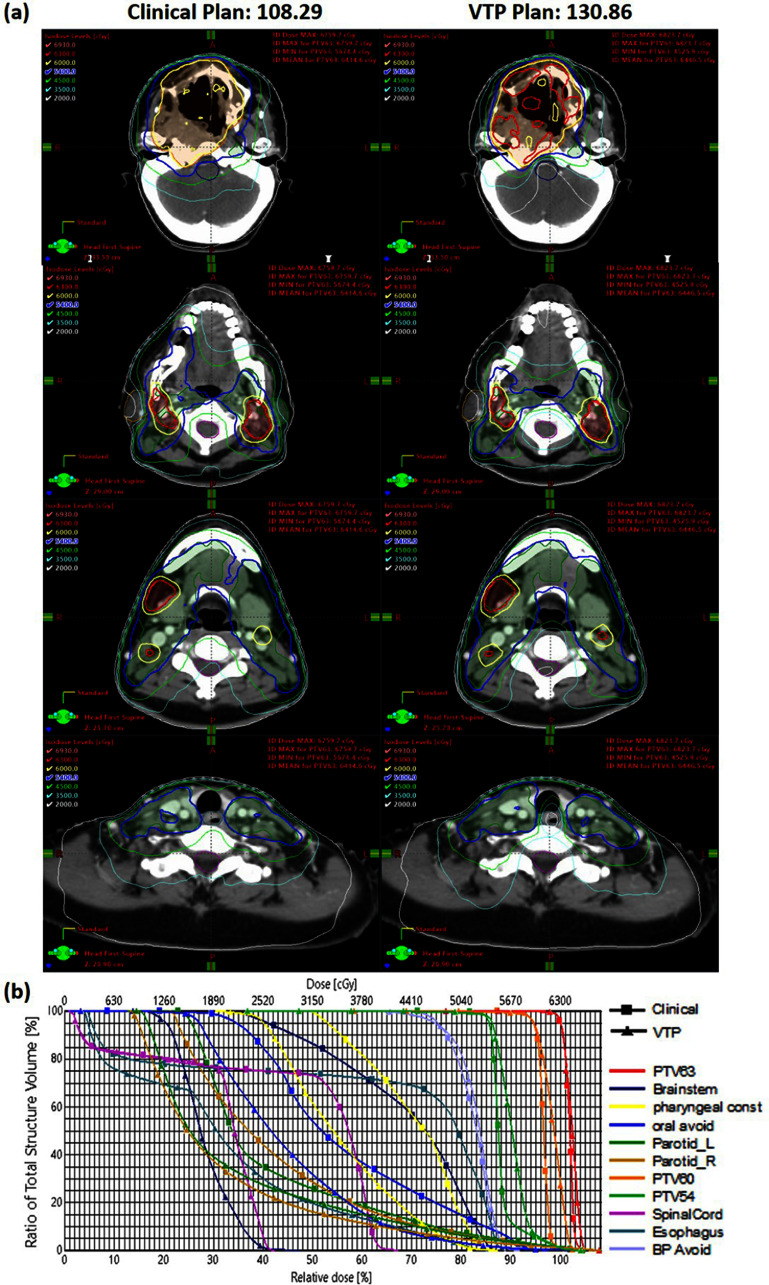
Comparisons between dose distributions, ProKnow scores, and DVHs of manual and VTP plans for a case example.

Figure 7 compares dose distributions at four axial levels, plan scores, and DVHs between VTP-generated and human-generated plans for a representative patient case. The VTP-generated plan presented superior dose sparing of OARs, including the brainstem, spinal cord, parotid glands, oral cavity, esophagus, and pharyngeal constrictor, without sacrificing the PTV coverage. The VTP-generated plan achieved a total plan score of 130.86, significantly outperforming the clinical plan, which scored 108.29. It is worthwhile to point out that the VTP-generated plan had lower homogeneity with larger hot spots in PTV60 and PTV57, which may be ascribed to the fact that the ProKnow criteria did not include this requirement, and hence VTP was not trained to achieve this. In contrast, the human planner prioritized homogeneous doses across all PTVs. Despite achieving a higher ProKnow score and better OAR sparing, the hotspots in the lower dose PTVs may not be clinically acceptable.

**Figure 8.**
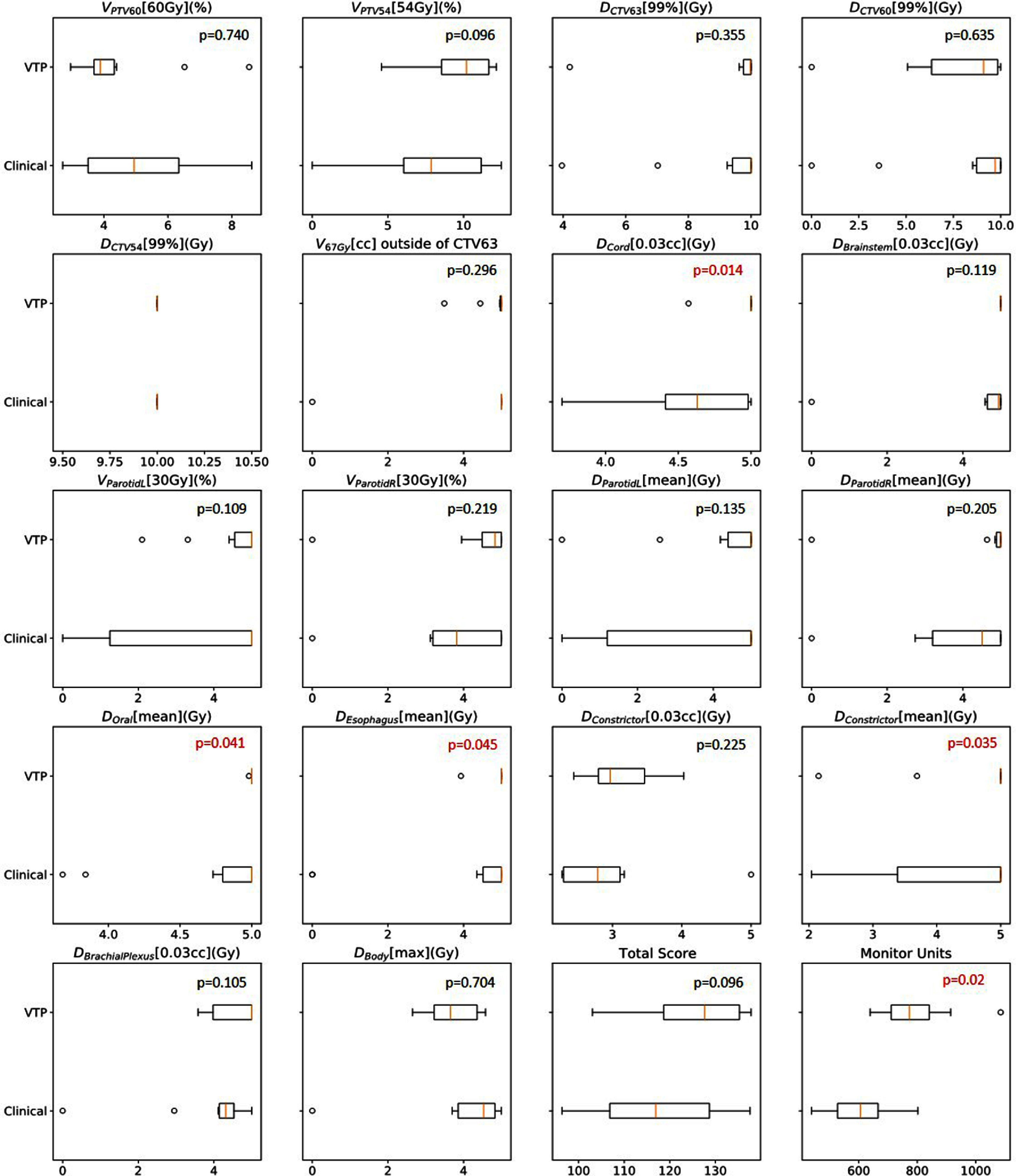
Boxplots display the ProKnow scores for clinically relevant plan quality metrics, the overall plan score, and monitor units, comparing 20 clinically approved plans and their corresponding ones generated by VTP. Median values are represented by horizontal lines inside the boxes, and the calculated p-value for non-inferiority testing is displayed in the upper right corner of each boxplot. The p-values below 0.05 are highlighted to indicate statistical significance.

Figure 8 summarizes the ProKnow scores for 18 clinically relevant dosimetric metrics measuring various aspects of plan quality, as well as total plan score, and MUs. We also performed a non-inferior statistical test for each metric to compare them. Statistical significance was considered with a threshold of $p^* = 0.05$. On the whole, VTP achieved an average plan score of $125.33\pm11.12$, in contrast to clinical plans scored an average of $117.76\pm13.56$ (*p* = 0.069). This indicated that the quality of VTP-generated plans was slightly better than those generated by human planners, although the improvement was not found to be statistically significant.

Overall, there was a trend that scores of plans generated by VTP were higher than the corresponding counterparts in the clinical plans. VTP outperformed human planners statistically significantly in several specific metrics, including $D_\mathrm{cord}[0.03cc]$ (*p* = 0.014), $D_\mathrm{Oral}[\mathrm{mean}]$ (*p* = 0.041), $D_\mathrm{Esophagus}[\mathrm{mean}]$ (*p* = 0.045), and $D_\mathrm{Constrictors}[\mathrm{mean}]$ (*p* = 0.035). Specifically, VTP-generated plans received scores of $4.97\,\pm\,0.12$, $5.00\,\pm\,0.01.56$, $4.90\,\pm\,0.32$, and $3.15\,\pm\,0.51$ for these four metrics, respectively. In contrast, clinical plans scored at $4.59\,\pm\,0.40$, $4.69\,\pm\,0.50$, $3.93\,\pm\,1.98$, and $4.21\,\pm\,1.05$.

VTP-generated plans exhibited higher MUs, with an average of $795.35\,\pm\,125.18$ MU per fraction, compared to human-generated plans with $607.43\,\pm\,108.16$ MU (*p* = 0.002). This implies that VTP-generated plans tend to have more intensity modulations and greater complexity. Considering that the prescription of the highest dose level was 210 cGy per fraction (6300 cGy in 30 fractions), the modulation factors defined as the ratio between MUs and prescription dose were ${\sim}3.8$ for VTP plans, and ${\sim}2.9$ for clinical plans, both lower than the empirically acceptable threshold of 4.0 to warrant deliverability of the plans.

The automated planning process proved efficient, producing plans in an average of $91.46\pm15.14$ minutes. VTP’s decision-making was nearly instantaneous, with the majority of planning time spent on Eclipse’s optimization process.

## 4. Discussions

### 4.1. About the results

In figure 7, we observed different plan characteristics between the clinical and VTP-generated plans, e.g. in target homogeneity, dose falloff, etc. The clinical plan exhibited a low plan score with moderate OAR sparing but a higher homogeneity for PTV60 and PTV57. In contrast, the VTP plan scored higher, demonstrating excellent OAR sparing but a poorer homogeneity. This fact can be ascribed to different planning objectives between the human planner and VTP. Notably, the PTV heterogeneity index was not factored into VTP’s plan optimization process, as it was not a criterion of the AAMD plan challenge upon which our optimization system was built. However, the ProKnow scores included the requirement for the overall hotspot (${\lt}$69Gy). Hence, VTP only considered the PTV dose homogeneity for the PTV63 to a certain extent, but not for the other two targets. In contrast, human planners aimed for plans with homogeneous doses across all PTVs, according to our institutional planning guidelines. It is also for this reason that the dose falloffs outside PTV boundaries were made shaper by VTP due to an attempt to spare normal organs allowed by sacrificing PTV homogeneity, compared to those in the human-generated plans. While this behavior of VTP was understandable, it in fact highlighted the need to clearly define treatment planning objectives for VTP to effectively execute planning and generate proper plans.

In figure 8, although the plan scores achieved by VTP were higher than those achieved by human planners, it is worth remarking that we present the results only for the purpose of demonstrating the capability of VTP for automated treatment planning by autonomously interacting with the TPS. Multiple reasons may cause the relatively lower scores of human planners, but the plans themselves are still acceptable for the clinical management of these patients. In particular, as the VTP was developed for the AAMD Planning Challenge and the Challenge evaluated the plan quality using the ProKnow score, the VTP incorporated this score in the reward function and hence was trained to learn to optimize this score. In contrast, the clinical plans we extracted for comparison were previously developed for clinical usage. Human dosimetrists developed them following our institutional planning guidelines to meet planning objectives specified by the attending physicians, which were not quantified by the ProKnow score. Hence, the VTP-generated plans and human-generated plans were developed under the guidance that was not necessarily aligned, which contributed to the different plan scores when evaluated using the ProKnow criteria. A higher ProKnow score does not necessarily guarantee clinical optimality. A related question here is whether a numerical metric may be defined to capture all aspects of plan acceptability, including both dosimetric quality and physician’s preference. Answering this question remains an area of ongoing research.

### 4.2. Contributions of this study

Compared with previous work, the contributions of this study were threefold. First, we achieved a fully automated IATP workflow for the clinically challenging scenario of H&N cancer RT treatment planning. The previous focus on prostate cancer represented a relatively simple treatment planning scenario, with only a few critical structures in immediate proximity to the prostate target, allowing for a relatively straightforward strategy to achieve effective dose coverage to targets and OARs sparing. In contrast, H&N cancer treatment planning is a much more challenging task, even for human planners, due to the unique dosimetric requirement and anatomical complexity. For instance, H&N cancer tumors can be in irregular shapes with large volumes and the plan has to simultaneously address multiple targets with different prescription dose levels. The proximity of the target to normal organs substantially increased planning complexity to achieve a sharp dose fall-off for normal organ sparing. Our study for the first time demonstrated the feasibility of addressing this intricate treatment planning problem using the DRL approach.

Second, we overcame the challenge of low training efficiency when using a real clinical TPS. Our earlier work in prostate SBRT trained the DRL agents using an in-house TPS similar to the commercial TPS. Integrating VTP to Eclipse during model training enhances generalizability and eliminates the issues caused by different dose engines between the in-house TPS and Eclipse. Previously for prostate SBRT cases, we were not able to use Eclipse due to the large amount of optimization tasks to be accomplished to train the VTP with a relatively large network size and a number of possible actions. It was acceptable to use the in-house TPS to train VTP and apply it to Eclipse, likely because of the relatively easy planning task for prostate SBRT and loose planning requirements. For H&N cases, the planning task is more difficult, and it is necessary to maintain consistency between TPSs used in model training and application. Hence, this study used Eclipse TPS for model training and to achieve so, we innovatively developed strategies such as reducing network complexity and the number of possible actions.

Third, the VTP was trained to address the 2023 AAMD Planning Challenge. As only one patient case was available for this competition, we trained VTP on this single case via the end-to-end DRL approach. Different from the majority of deep learning studies that are inherently limited by available data, DRL generates data by itself during the training process. Our study demonstrated that the VTP trained on only one patient achieved great generalizability in terms of performing high-quality treatment planning for real patient cases not seen in the training process.

### 4.3. Limitations and future work

**Figure 9.**
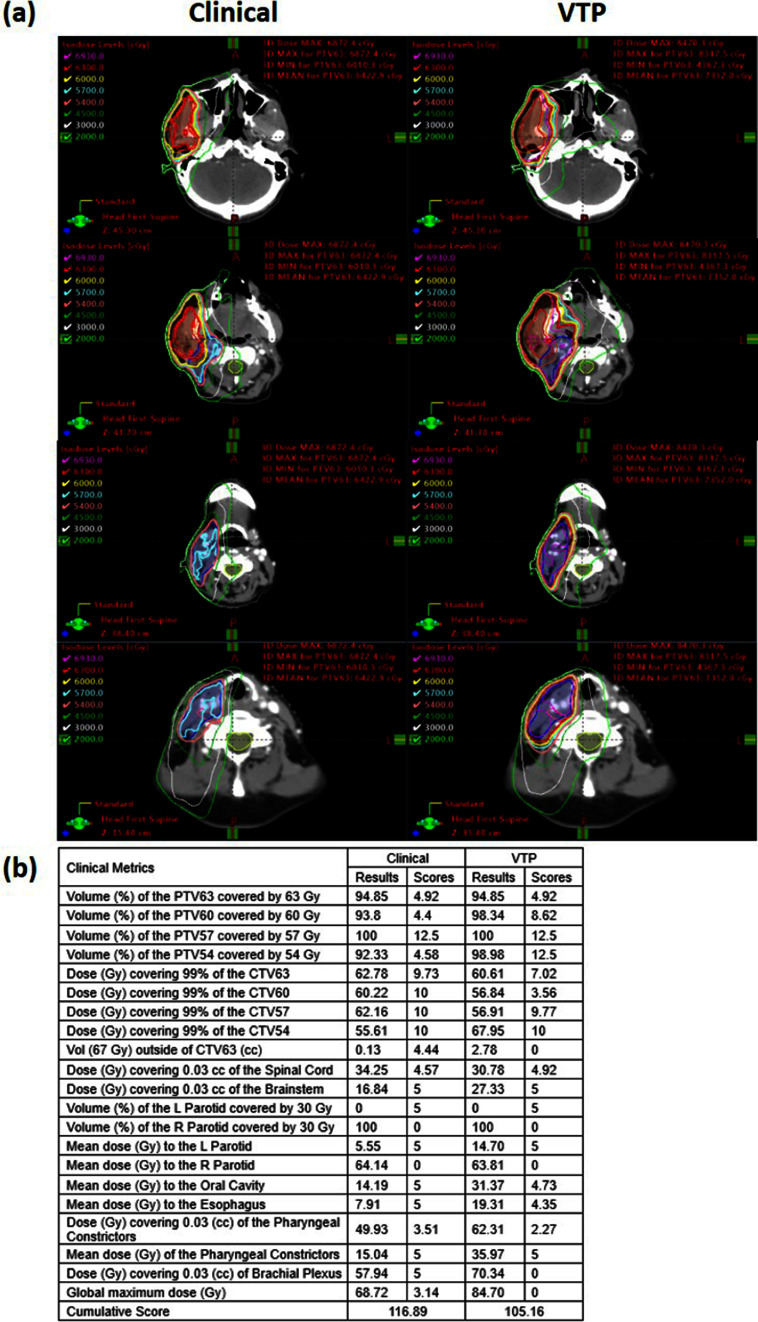
Comparisons between dose distributions and ProKnow scores of manual and VTP plans for a challenging case for VTP.

The current study has the following limitations. First, model generalizability is a central topic of deep learning. When characteristics of the data at the model inference stage deviate from that of the training data, model performance may degrade. DRL training is less restricted by the quantity of training cases, as the training data is in fact state-action pairs generated during the training process itself. In this study, we successfully demonstrated this aspect by showing that VTP trained on a single patient case can still generally perform relatively well in other real clinical cases. Nonetheless, we remark that the fact that the data were generated for only one patient still bears the generalizability concern. We observed that VTP encountered difficulties in cases that have quite distinct anatomy from the case used in training. For example, during comprehensive evaluation, a difficult case was observed where VTP performed notably worse. This patient underwent unilateral neck RT, with PTV volumes substantially overlapping with the brachial plexus. As VTP had not encountered such a scenario during training, it struggled to balance target coverage and OAR sparing, as shown in figure 9. Therefore, it is still desired to further train the VTP in other cases to enhance its generalizability, which is our ongoing work.

Second, the VTP was trained to only adjust TPPs in inverse planning optimization. However, other parameters may significantly influence the resulting plan quality and should be adjusted as well. For example, the collimator angle may affect dose fall-offs around the targets. Human planners know to set up parameters like this based on experiences. Our current study represents just the initial phase in developing the IATP framework for challenging H&N cases. We expect extensive subsequent studies to enhance the intelligence level of the virtual planner.

Third, the simplicity of the ProKnow scores may not fully capture the complexities of planning objectives in real clinical practice. As discussed previously, to align with our institutional treatment planning guideline, target homogeneity should be included. Moreover, recognizing the limitations of quantitative metrics, integrating criteria such as physician judgment is necessary to ensure clinical acceptance of VTP-generated plans. One potential solution involves leveraging our recent development of a deep learning-based virtual physician (Gao *et al* 2022, 2024), which predicts plan approval probability using adversarial learning based on clinically approved plans. We plan to build the virtual physician model to the H&N context and integrate it into the DRL training in our future study. Doing so is expected to generate plans that not only meet dosimetric standards but also closely align with the clinical preferences of human physicians. On the evaluation side, in addition to quantitative measure plans with certain metrics, it is also important to involve domain experts to fully assess the clinical impact of our research development. We are in the process of performing human evaluation studies comparing VTP plans and human-generated plans and commenting on their strengths and weaknesses. The results will be reported in our future publication.

## 5. Conclusion

In this paper, we reported our recent progress on the continuous development of the IATP framework by extending the VTP to H&N cancer treatment planning, a much more challenging task than prostate cancer treatment planning addressed by our previous studies. We implemented a hierarchical DRL framework for the VTP to mimic the treatment planning processes performed by human planners. We seamlessly integrated VTP with the Eclipse TPS with ESAPI to train VTP using this TPS and achieve a fully automated treatment planning workflow. In a prospective evaluation context of the 2023 AAMD Planning Challenge, the VTP achieved first place in treatment planning efficiency. An evaluation study using 20 real clinical cases showed that the VTP achieved an average score of $125.33\pm11.12$, higher than that of the plans generated by experienced human planners at $117.76\pm13.56$. These results demonstrated the potential of VTP for automated H&N cancer treatment planning.

## Data Availability

The data cannot be made publicly available upon publication due to legal restrictions preventing unrestricted public distribution. The data that support the findings of this study are available upon reasonable request from the authors.
